# Dietary Supplements: A Gateway to Doping?

**DOI:** 10.3390/nu15040881

**Published:** 2023-02-09

**Authors:** Moriah Mallick, Chloe Briana Camacho, Jana Daher, Dalia El Khoury

**Affiliations:** Department of Family Relations and Applied Nutrition, University of Guelph, 50 Stone Road, Guelph, ON N1G 2W1, Canada

**Keywords:** dietary supplements, doping, contamination, athletes, sports

## Abstract

Dietary supplements are widely used among athletes, but many may be unaware of the potential for unintentional doping, especially considering that supplements can often be contaminated with prohibited substances. The reason behind the widespread use of dietary supplements among athletes is often cited as being for the purposes of enhancing health or performance. However, many athletes turn to unreliable sources of information, and often lack knowledge of dietary supplement regulations. The aim of this narrative review is to explore the current research surrounding the psychological constructs (such as norms, attitudes, and beliefs) related to dietary supplement use among athletes that may lead to inadvertent doping. This review also covers possible programme structures that may be effective at preventing inadvertent doping.

## 1. Introduction

The World Anti-Doping Agency (WADA) is an independent international organisation based in Canada. Since 1999, WADA has been striving to regulate fair play in sports by producing anti-doping rules and practices to lead the global movement for doping-free sport. This includes obligatory drug testing in athletes competing under an anti-doping code [1]. To maintain integrity in sports and to safeguard athletes, WADA publicly shares a list of prohibited drugs and methods (e.g., gene and cell doping, the manipulation of the blood, etc.) each year [2]. WADA strictly states that athletes are responsible for everything they ingest or put into their bodies [3]. Due to these strong liability provisions, even if an athlete is found to have unintentionally used a prohibited substance, WADA’s code mandates that an adverse analytical finding be recorded [4]. Not only can this result in a temporary or permanent ban from competing, as well as the potential revocation of past winnings or records, it may also cause a great financial burden to the athlete due to financial penalties, loss of employment, and loss of sponsorships [4].

The unintentional use of a prohibited substance is called inadvertent doping. According to a clinical review, the main causes of inadvertent doping are negligence and the misuse of dietary supplements (DS) [2]. DS are commercially available products intended to supplement one’s diet to promote or maintain good health [5]. Examples of DS are vitamins, minerals, probiotics, proteins, herbs, and botanicals [6]. Many DS claim to provide favourable effects including weight loss, increased energy, accelerated recovery, increased cognitive and physical performance, and overall improvements in health [6]. However, it is critical to understand that most of the claims made about DS are unproven, and that there are no defined standards for the quality of the evidence required to support such claims [7,8]. For example, studies with numerous design and experimental flaws may still be presented as having sufficient scientific evidence to back a claim made by a DS company [8]. Therefore, it is not uncommon for scientific evidence purporting the usefulness of DS to emerge from the least rigorous studies [4]. In contrast to pharmaceutical products, which are subject to strict pharmaceutical rules based on premarketing authorisation and end-product supervision, DS manufacturers and distributors are also not required to obtain Food and Drug Administration approval before selling them. Countries regulate DS on a national level, and regulations vary from one nation to another [9]. Evidently, DS regulation is lax; there are no global quality control rules, which explains why there are several substandard products throughout the world.

Moreover, many athletes are under the impression that if a DS is on the market, it must be safe; however, that is not always the case. Since DS fall under a subcategory of food, DS producers are not required to show that their products are safe and effective before marketing them [9]; therefore, DS producers are not obligated to test their products for any of the prohibited substances listed by WADA [9]. A meta-analysis examining the prevalence of contamination in DS found that almost 28% of the examined supplements posed a potential risk of inadvertent doping [10]. They also found that 875 out of 3132 examined DS contained undeclared substances [10]. This increases the likelihood of experiencing adverse health effects [9]. A recent review that explored the reported prevalence of dietary supplement use among athletes found that the prevalence was greater than 50% in the majority of the selected studies [11]. Therefore, it is vital that athletes understand the risk of consuming DS, as trace amounts of prohibited substances can still result in a failed drug test even in quantities that are too low to have any actual physiological effects [4]. A thorough risk analysis should be conducted by athletes to determine whether the possible marginal improvements would be worth the chance of contamination-related inadvertent doping [4].

Interestingly, DS use also significantly correlates with intentional doping in sports [2]. Studies have found that DS users show more favourable behaviours and positive attitudes towards doping [2,12]. In certain ways, DS may be considered as a slippery slope to intentional doping, lending credence to the gateway hypothesis. The term “gateway hypothesis” is typically used to describe the likelihood that the adoption of a certain behaviour, which is considered safe (in this case, DS use), could then result in the subsequent adoption of another behaviour that is considered harmful (in this case, use of prohibited substances) [13]. Therefore, it can be hypothesised that internalising and normalising regular use of DS may serve as a gateway to the abuse of prohibited substances in sports. First, an athlete may try performance-enhancing supplements to gain a slight advantage over their competitors; next, they may start experimenting with some harder drugs [14]. Considering a study which found that DS users had a three-and-a-half times higher prevalence of doping usage compared to those who didn’t use DS, the gateway theory is worth investigating [2].

Our review aims to explore the psychological constructs (including the attitudes, norms, and beliefs of DS athlete users) that may lead to inadvertent doping. Articles published in the last 40 years are included in this review.

## 2. Predictors of Doping

There are many factors that predict doping behaviour in athletes, and the relationships are often complex. In order to prevent the risk of doping, it is important to identify these predictors so they can serve as a foundation for education or intervention programmes. Dietary supplement use has been cited as a major predictor of doping behaviour, which is supported by the fact that dietary supplements can often be contaminated with prohibited substances [10,15,16,17].

Demographic factors may also play a role in predicting doping behaviour. A systematic review found that males are at an increased risk of doping than females, and that age and doping intention were positively correlated, though this effect was small [15]. Sex as a predictor for doping exhibited equal results among young athletes, but the evidence for age as a predicting factor for doping was mixed [18]. Doping risk may also vary depending on the sport that an athlete participates in, with power or speed-based athletes having a greater doping risk than motor-skills-based sports, and individual sport athletes having a higher doping risk than team sports athletes [19,20].

A meta-analysis by Ntoumanis et al. (2014) analysed the theory of planned behaviour constructs in relation to doping and discovered that perceived social norms and positive attitudes towards doping were strong positive predictors of doping intention and doping behaviour. On the other hand, self-efficacy and morality were found to be negatively associated with doping intention and doping behaviour [15], with one study [21] finding that these two factors mediate the predictive effects of a coach’s perception of an athlete’s susceptibility to doping affecting likelihood of doping. However, a different meta-analysis by Blank et al. (2016) reported contradictory results in that intention was not found to be a significant predictor for doping behaviour, making it unclear as to what the relationship between doping intention and doping behaviour is [22]. This meta-analysis cited situational temptation, attitudes, and beliefs to be the strongest predictors of doping behaviour [22]. It is notable that this meta-analysis only included studies conducted on elite athletes due to the potential differences between recreational and elite athletes, each experiencing a distinct set of environmental pressures that mediate predictors of doping [22]. With many studies focused on both athlete-centred and individual predictors, they suggested that future studies ought to examine predictors for doping at a macroscopic level (i.e., sport culture) [22].

## 3. Contamination of Dietary Supplements, and Risk for Athletes

WADA defines prohibited substances as being prohibited at all times, both in and out of competition contexts. The list includes anabolic androgenic steroids (AAS), erythropoietin (EPO), peptide hormones and releasing factors, growth factors and growth factor modulators, beta-2 agonists, hormone and metabolic modulators, diuretics, and masking agents [23] (see Table 1 for some examples). These prohibited substances are often found in dietary supplements, and their use by athletes thus poses a high risk of unintentionally obtaining a positive doping test in their sports [10,16,17].

DS can often be contaminated with prohibited substances. A study found that out of the 3132 DS they analysed, there was approximately a 28% risk of unintentional doping due to the contamination of the supplement [10]. This was due to either the banned substances not being listed on the labels, or to the actual ingredients or amounts being different than what was listed on the product [10]. Sibutramine, followed by testosterone and other anabolic steroids, was the substance detected the most among the dietary supplements studied [10]. A study, reporting on studies from 12 countries focused on dietary supplement use in sports, found a contamination rate of 12% to 58% [17]. Given that athletes are known to be frequent consumers of DS, the prevalence of contamination in these products poses a significant risk to them [11,24].

The cause of dietary supplement contamination can be traced back to the poor-quality control involved in the production of these products [25]. Some evidence even points to deliberate adulteration of these products, though there is a lack of clarity on the scope of this issue as dietary supplements do not undergo comprehensive testing programmes [26]. One study found over-the-counter drugs to be at higher risk for unintentional doping than ethical drugs due to the contamination of ephedrine and its derivatives in cough remedies [27]. Not only does a positive doping test damage an athlete’s reputation and livelihood, but there are also such health risks as toxicity associated with consuming doping substances [28,29]. A review covering anabolic–androgenic steroid use, for instance, found that these steroids were associated with adverse cardiovascular effects (including cardiomegaly, fibrosis, and the necrosis of the myocardial tissue) in athletes who had any kind of history of anabolic-androgenic steroid use [30]. As such, the consequences can be dire for an athlete who unknowingly consumes contaminated dietary supplements. Even more importantly, it is crucial that efforts are made to regulate the quality and safety of DS.

## 4. Motivations of Dietary Supplement Use

**Figure 1:**
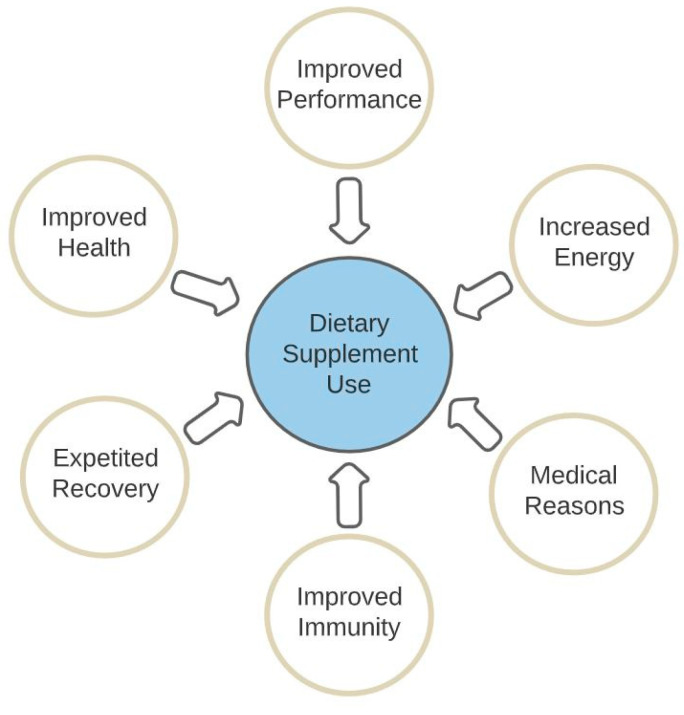
Athletes’ motivations for dietary supplement usage.

Considering that athletes worldwide are major users of dietary supplements and that this can be a strong predictor for doping, it may be helpful to understand the reasoning and motivations behind why athletes consume nutritional supplements in the first place (Figure 1). A study surveying young Canadian athletes found that 81% used dietary supplements to stay healthy, with ‘increasing energy’ and ‘improving the immune system’ as the next two most cited reasons for use, cited by 55% and 52% of respondents, respectively [24].

Similar results on the most cited motivations for DS use have been found worldwide, with the improvement of performance, health, and recovery being the most frequently cited reasons [11]. In addition, females were found to be more likely to use DS for health reasons, while males more frequently reported DS use for performance enhancement [11]. In professional Saudi Arabian athletes, results showed that 43.8% used DS to enhance performance and 32.6% used them for health benefits [31]. In national-level Sri Lankan athletes, by contrast, 79% consumed supplements for performance improvement, and 19% for enhancing health [32]. A systematic review examining actual dietary supplement use by athletes reported the primary reasons for use were for performance enhancement, recovery, and health maintenance [6]. A study examining elite Paralympic athletes also showed identical trends, with the top three motivations for dietary supplement use reported to be health, energy, and medical reasons [33].

There appears to be a common theme of athletes consuming dietary supplements with the motivation of enhancing their health and improving their performance, as these are consistently ranked as the top reasons for use. This is a cause for concern, considering that dietary supplements can be contaminated with doping substances, and that athletes’ information on supplements can come from unreliable sources or be obtained without medical supervision. It is also important to consider that the prevailing perception of dietary supplements as improving performance and health may not be entirely true as a large number of these claims are neither based on scientific evidence nor proven to be correct. Interestingly, a study by Paulsen et al. found DS produced undesirable results as opposed to beneficial effects on strength training [34].

## 5. Perceptions of Dietary Supplements and Doping

In addition to understanding the predictors of doping, it may also be useful to understand how athletes view dietary supplements and their relationship to doping behaviour when developing anti-doping programmes and educational materials. In a study focusing on athlete users, a greater belief in dietary supplements as being necessary for performance was associated with a higher likelihood of doping [35]. Furthermore, the indirect relationship between dietary supplement use and doping behaviour was negated when moral identity and values were low or moderate [36]. The type of supplement an athlete consumes may also affect this indirect relationship between dietary supplement use and doping attitudes via dietary supplement beliefs. One study observed this relationship among those who consumed sports foods and drinks, medical supplements, and ergogenic substances, but not among superfood supplement users [37].

As mentioned, many athletes’ motivation for dietary supplement use is to enhance health and performance [38]. In one study, most athletes cited it as a good alternative to illegal performance-enhancing substances [12]. The same study found that 76% of 212 competitive athletes would take nutritional supplements if it were to guarantee them a win, with a greater willingness found in regular users than non-users [12]. This study also showed that regular dietary supplement users demonstrated attitudes towards doping behaviour that were more positive than those of non-users, accompanied by a greater belief in doping’s effectiveness [12]. Dietary supplement users exhibiting a more positive attitude towards doping are likewise reported in Hurst et al.’s study, except that the differences were only found between non-users and users of ergogenic aids and medical sports supplements, and not between non-users and users of superfoods and sports foods/drinks [37]. Surprisingly, a study showed that most athlete participants perceived DS as containing doping agents, and as improving strength, energy, and endurance, but disagreed with the statement that it improved health [39]. Another study also found that a majority of athletes recognise that doping is risky, unhealthy, and a form of cheating [38].

## 6. Awareness of Dietary Supplement Regulations among Athletes

DS are not as tightly regulated as prescribed medications are. Since DS are classed as a subcategory of food, manufacturers are not required to produce evidence of their products’ safety or efficacy [9]. In the United States, the FDA oversees dietary supplement quality, safety, and labelling, while the Federal Trade Commission supervises commercials and marketing. Nonetheless, significant enforcement issues exist, and optimal regulatory control has not been attained. If the composition and quality of substances cannot be consistently guaranteed, the validity of dietary supplement research is called into question [8]. Similarly, the Canadian DS sector have developed a number of regulatory standards to ensure safety, but these requirements have not always been correctly implemented [40]. According to the Government of Canada, the growth of the DS market has made it difficult for government regulators to closely monitor every product that enters the market [41]. Since DS regulations vary by country, there are supplements available in illegal markets that are labelled as dangerous, and do not fulfil the required quality criteria [42]. For example, the Government of Canada acknowledges that the Internet makes it simple for Canadians to buy these products from other countries. One study found the internet to be a preferred site for DS purchases among elite Spanish athletes [43]. This ease of access to tainted DS and “black market” items poses a threat to public health; due to the lack of consistency and limited regulation of DS across the globe, especially in DS purchased outside of Canada, DS safety cannot be guaranteed [41].

This raises the question as to whether athletes are aware of the laws and regulations governing DS in their country. Athletes must be mindful of the numerous problems that may result from the lax regulations surrounding DS if they are to make informed decisions about consuming DS. Many studies have revealed the limited knowledge among athletes and the general public of their governing bodies’ DS regulations and anti-doping guidelines [44,45]. For example, one study found that only 55.5% of young athletes both had access to and were aware of DS regulations [45]. Similarly, none of the 170 adolescent recreational athletes polled in another study stated they were aware of the regulations governing DS [46]. Shockingly, these findings are not uncommon. Several other studies have also found many American college students, adults, senior citizens, and doctors are also unaware of the FDA’s minimal participation in the regulation and testing of DS [47,48,49,50,51]. To ensure the safety, efficacy, potency, and legality of DS, it is necessary to reform the current regulations controlling the market for DS, and thus protect the public, especially athletes, from the adverse effects related to DS use and inadvertent doping [17]. It is essential that athletes choosing to consume DS are aware of the limited involvement of current DS regulations so they can protect themselves against anti-doping violations.

## 7. Sources of Information

Given the prevalence of dietary supplement usage among athletes, it is crucial to be aware of the sources from which these individuals are learning about dietary supplements and dietary supplement use. This is due to the several risks associated with dietary supplement misuse, including inadvertent doping and adverse health effects. Athletes who receive information from untrustworthy sources are more likely to suffer detrimental impacts due to a lack of knowledge.

Numerous studies have found that athletes’ primary sources of information on DS are their coaches, teammates, the internet, or their relatives and friends [11,24,40,45,52]. For example, one review found that coaches have the largest influence on DS use in athletes, trumping the influence of doctors and sport dietitians [9,45]. These findings are consistent with another study which found that family and friends were the primary sources of knowledge (74%), followed by coaches (44%), athletic trainers (40%), doctors (33%), and finally sports nutritionists (32%) [53]. Additionally, another review found an association between sex and sources of information [11]. They found that males were more likely to turn to their coach or trainer, their teammates, their dietitian or nutritionist, and their family and friends for DS information. By comparison, females were more likely to turn to doctors or other healthcare providers, their coach or trainer, and their family and friends [11]. This finding does not apply to the studies that did not find an association between sex and sources of information, but it is important to note that this finding does contradict another study that only found significant associations between sources of information and type of sport and age, not sex [40]. They found that younger athletes referred to their trainers more frequently than older athletes for information about DS, and that endurance athletes referred to health professionals for DS information more frequently than power and intermittent sport athletes [40].

Evidently, the sources of information athletes utilise are unreliable and of low quality [2]. Given the likelihood of DS contamination with illegal substances, subsequent risk of anti-doping punishments, and the potential ensuing health concerns associated with DS misuse, consulting a trustworthy source of information about DS is essential in the sports world. A possible way to reduce the likelihood of negative DS experiences among athletes would be to ensure their sources of information are educated about DS products, dosages, and uses, as well as the risk of contamination that comes with DS use. Accordingly, sports dietitians and nutritionists play a critical role in educating athletes and coaches about nutrition and how to plan their diets to maximise performance and recovery [54]. It is critical that the coaches and trainers who provide DS knowledge have an adequate DS education, and that they follow the suggestions of scientists and the available scientific material [45,54]. There is an urgent need for a more thorough education on these products as sports supplement consumption rises [45].

## 8. Third-Party Testing

Another way athletes can protect themselves against inadvertent doping is by using third-party certified dietary supplements. Third-party testing organisations assist in the regulation of dietary supplements by evaluating their quality, purity, efficacy, and composition before retail distribution [2,55]. Once approved by the third-party programme, the dietary supplement receives a “seal of approval” that consumers may look for before purchasing. As the market grows and dietary supplements become utilised more frequently, the demand for third-party verification increases [56].

To obtain third-party certification, the dietary supplement must be examined by a neutral body with an understanding of quality assurance to ensure it fulfils a set of standards. Therefore, for a company to be recognised as third party, it must not be affiliated with the supplement company seeking certification and must also have no government regulatory authority [57]. This approach is thought to provide a key level of transparency to consumers and greatly reduce the likelihood of contamination. However, it is also important to note that since it is difficult to test for every prohibited substance, third-party programmes cannot assert that a product is completely free from all banned substances even after certification testing; nevertheless, third-party certification still provides solid evidence of a reduced risk of contamination, despite not being able to entirely remove the possibility of a contamination of some sort. The involvement of third-party testing is completely voluntary, and no dietary supplement producers are required to undergo third-party testing before marketing their products [2]. Despite the potential benefits of third-party testing, the use of these organisations and their certified products is not widely reported among athletes [55]. This study found that, despite over 90% of participants acknowledging the value of acquiring third-party certified supplements, only 57% said that their supplements had been third-party tested [55]. Given the serious consequences of contaminated supplements on an athlete’s career, using third-party tested supplements may be a great strategy to significantly reduce the likelihood of inadvertent doping. Athletic trainers must also be knowledgeable about third-party testing programmes in order to ensure proper dietary supplement use [56]. Finally, it is important to acknowledge the role that governments should play in accrediting and overseeing the testing procedures implemented by these third-party testing bodies.

## 9. Prevention: A Focus on Education

The widespread use of DS and the numerous contributing factors (such as sources of information, awareness of dietary supplement regulations, perceptions of doping and DS use, contamination of DS, and motivations for DS use) indicate that intervention education programmes designed to focus on improving the knowledge, beliefs, and attitudes towards DS use may be an excellent first line of defence against inadvertent doping. Many reviews focusing on DS use in athletes have found compelling evidence in support of interventional education programmes for athletes (and young athletes, especially), coaches, and parents/family members [9,11,53,58].

A narrative review focusing on educational interventions to advance understanding, attitudes, behaviours, and practices about DS and doping substances found consensus among the studies, proving educational intervention to be effective at increasing knowledge of DS and doping substances among participants [58]. The majority of the studies in this review delivered their curricular materials through a variety of methods. The most commonly reported intervention procedures and modes of delivery were lectures and demonstrations, group conversations, and written materials such as flyers, handouts, posters, brochures, and booklets. All studies, with the exception of three, conducted face-to-face interventions. Some examples of providers of information in these studies included the researchers themselves, trained staff members, physical education teachers, coaches, track and field athletes, team leaders, and health professionals. The duration of interventions amongst the reported studies varied, with some lasting only five minutes and others lasting up to two years [58].

With regard to attitudes and intentions, the narrative review found that, out of the twenty-five studies analysed in their review, ten reported improvements in the participants’ attitudes and intentions towards the use of doping agents [58]. However, interestingly enough, some experts believe that drug education can have unintended consequences, similar to how changing attitudes regarding doping can [59,60]. A probable rationale for instances of increased drug use or favourable views towards drugs following educational intervention is the psychological reactance theory [60]. Reactance may occur when an individual believes their freedom of choice is being curtailed by an external agent [61]. As a result, individuals may seek to regain control of their actions by acting in the opposite direction of the desired behaviour.

The number and diversity of athletes, along with corporate marketing and false information in the media, pose challenges to improved DS-related education [53]. A Canadian study found that athletes suggested that they would respond to broader instructional media, such as the internet, e-mail, social media, and presentations given to larger groups of people [53]. Therefore, they suggest online courses, computer/video games, and videos may all be effective educational tools to improve DS-related knowledge, attitudes, and beliefs in athletes [53]. Overall, it is imperative that sports organisations, especially universities and colleges, educate their athletes and coaches about DS in order to prevent both inadvertent and intentional doping among their athletes.

## 10. Gaps

Additional research is needed regarding the development of DS-related educational programmes designed to prevent inadvertent doping in athletes, as well as into the effectiveness of such programmes. Integrating theory into these programmes may prove to be beneficial, considering the influence that knowledge, norms, and attitudes have on doping in sports. Moreover, more rigorous regulations are needed to ensure the safety and purity of DS, and to protect general health against the consumption of undeclared or contaminated substances. Additional research should also be conducted to identify the extent of athlete engagement with reliable sources of information, and the accessibility of such resources, from health professionals to athletes.

## 11. Conclusions

In conclusion, contaminated dietary supplement use is a pressing issue, considering it is a major predictor for doping. Perceived social norms and positive attitudes may both be constructs worth targeting when considering doping prevention. There is also a great lack of awareness regarding DS regulations among athletes, and in addition, they often turn to unreliable sources of information when it comes to DS use. A commonly held perception athletes have about DS use is that it can enhance health and performance, but this may be a misconception and can end up becoming detrimental to their livelihood, reputation, and health. It is essential that a multifaceted approach is taken to ensure the safety of athletes when it comes to dietary supplements. Reforming the regulation controls surrounding DS production is a good starting point. In addition, sports organisations, sports dietitians, trainers, and coaches also have great influence and crucial roles to play in educating athletes on dietary supplement use. Lastly, athletes must exercise vigilance over what they consume, and likewise take the necessary precautions to ensure they do not inadvertently dope.

## Figures and Tables

**Table 1:** WADA’s Classification of prohibited substances, with examples [23].

Prohibited Substance Classification	Examples (Included but Not Limited to)
Anabolic agents	Testosterone, dehydroepiandrosterone (DHEA), androstenediol, drostanolone
Peptide hormones, growth factors, related substances and mimetic	Erythropoietin receptor agonists, chorionic gonadotrophin (CG), growth hormone releasing factors, growth hormone (GH), insulin-like growth factor (IGF-1)
Beta-2 agonists	Arformoterol, procaterol, terbutaline, higenamine
Hormone and metabolic modulators	Testolactone, letrozole, clomifene, tamoxifen, myostatin-binding proteins, trimetazidine
Diuretics and masking agents	Desmopressin, acetazolamide, furosemide, spironolactone, thiazides

## Data Availability

Data sharing not applicable.
